# Barriers to Initiating Psychotherapy Faced by Jewish-Identified People in the United States

**DOI:** 10.1007/s10943-024-02097-2

**Published:** 2024-08-16

**Authors:** Anna Jean Berman, Scott Woolley

**Affiliations:** Alliant International University, 10455 Pomerado Road, San Diego, CA 92131 USA

**Keywords:** Jewish, Barriers, Psychotherapy, United States

## Abstract

The researchers investigated stigma against psychotherapy, Jewish culture, Rabbinical influence, and religiosity as perceived barriers by Jewish persons in the United States when initiating psychotherapy (n = 94). Researchers discovered stigma as a barrier (*p* = 0.015). Age, location, gender, and relationship status were added as predictors, revealing male gender (*t*(84) = 6.257, *p* < 0.001) as a negative predictor, and Rabbinical influence (*t*(84) = 2.049, *p* = 0.044) as a positive predictor for initiating psychotherapy.

## Barriers to Initiating Psychotherapy Faced by Jewish-Identified People in the United States

Religion plays an important function in the lives of clients and societies (Anderson, 2015). Consequently, therapists need to understand how religion impacts the behaviors and mental health of clients and their social systems. Ethical standards obligate therapists to address spirituality and religion, yet there is a lack of knowledge and training on religiosity and spirituality (Ginsberg & Sinacore, 2013; Vieten et al., 2016). Most religious research has focused on North American, white, Christian samples (Levin, 2013). Little research exists on minority religious groups, and research focusing on Jews or Judaism is even more limited (Levin, 2013; Weinrach, 2002). There is evidence of an increasing interest among Jews in attending therapy (Ginsberg & Sinacore, 2013); however, we do not understand what barriers exist for Jews in need of psychotherapy. More research on the intersection of Jews and mental health would benefit the field and the Jewish community since diversity literature still either does not include Jews or does not recognize Jews as having a culturally distinct identity (Langman, 1995; Weinrach, 2002). The purpose of this study was to discover perceived barriers faced by Jewish-identified persons in the United States of America when initiating psychotherapy.

## Literature Review

The religion of Judaism is a monotheistic, Abrahamic religion, based on the Torah and the Rabbinical Talmud (Miller & Lovinger, 2000; Rabinowitz, 2000). The Torah and the Talmud contain laws for the Jewish people to follow as the “chosen” people of God (Miller & Lovinger, 2000; Rabinowitz, 2000). Two percent of Americans identify as Jewish, and 14 million Jews live around the world (Walsh, 2010). Furthermore, many Jews identify as culturally Jewish, but not as religiously Jewish (Miller & Lovinger, 2000; Mitchell, 2021). There are three main types of Judaism in the United States that different subgroups of Jews fall under: Orthodox Judaism, Reform Judaism and Conservative Judaism.

Orthodox Judaism is strict adherence to the Torah and Talmud (Margolese, 1998). Those who follow Orthodox Judaism also follow the Halchot, which are a collection of additional guidelines that pertain to aspects of daily living such as business transactions, dietary laws, sexual behavior, and family relations (Margolese, 1998). Although strict, there is a spectrum of Orthodox Judaism subgroups (Schnall, 2006). There is Modern Orthodox, Yeshiva Orthodox, Chabad, and Hassidic Jews (Krumrei et al., 2013). Most Orthodox Jews are named after a particular dynasty of Rabbinical leaders (Margolese, 1998).

Reform Judaism began in Germany as part of the Zionist movement (Miller & Lovinger, 2000; Rabinowitz, 2000). Reform Judaism focuses more on the humanistic movement and civil rights happening in the nineteenth century and ignores more ritualistic traditions of Orthodox Judaism (Miller & Lovinger, 2000).

Conservative Judaism started as a reaction to Reform Judaism to incorporate more of the tradition of Orthodox Judaism. Conservative Judaism started in the United States and those that adhere to Conservative Judaism attempt to follow more traditions while not being as rigid as the Orthodox beliefs (Miller & Lovinger, 2000). Knowing how different kinds of Jews follow the religion is necessary for clinicians because Jewish beliefs can influence the type of therapy or the kind of therapist a Jew searches for when they initiate therapy.

## Barriers to Psychotherapy Faced by People of Faith

Richards and Bergin (2005) wrote that historically, behavioral sciences and religion have been “alienated” from each other (p. 6), causing researchers of psychology to ignore the influences of religion on therapy. Even the leaders of psychology, such as Freud, deliberately advocated against incorporating the spiritual and religious into psychology (Richards & Bergin, 2005). This exclusion led to the needs and beliefs of religious clients to be ignored (Richards & Bergin, 2014). Over time, this added to the distrust religious clients had against mental health professionals because those professionals had advocated for values and practices that conflicted with religious communities over the past century (Richards & Bergin, 2014). Vieten et al. (2016) surveyed 222 psychotherapists and licensed clinicians and found a general “lack of training” (p. 9) in religious diversity issues.

People from marginalized faiths tend to face barriers to accessing psychotherapy services. For example, Haque and Tubbs (2019) found in their mixed methods study of 314 Muslims in the United States that Muslims have similar stress levels as other minority groups, with the major source of distress coming from perceived micro-aggressions. Muslims experiencing micro-aggressions might not be willing to seek out therapy for fear of discrimination or covert Islamophobia. The authors wrote that clinicians could help diminish micro-aggressions by educating themselves on Islam and seeking out support and information from the Muslim community itself. Because of ignorance and micro-aggressions, Muslims may prefer to rely on their religious leaders and faith tenets to solve problems. Therefore, reliance on religion and the influence of their culture might be a barrier to therapy for Muslims.

Lyon (2013) found that Latter-day Saints have tended to rely on their religion to help them find solutions to problems or psychological issues instead of turning to the psychology profession. They may view turning to a therapist for answers as proof that they are not devout to their God and their religion. In fact, the Latter-day Saints’ reliance on their religious leader could sometimes mean delaying psychotherapy when it could be beneficial because they would rather turn to the religious leader. Finally, a barrier to psychotherapy is the influence of Latter-day Saints’ culture on the members to hesitate to talk to therapists because they may believe that therapists are more likely to pathologize their beliefs, rituals and traditions (Lyon, 2013). Due to fear of being misunderstood and fear of prejudice, Latter-day Saints face barriers to psychotherapy that include reliance on religion, reliance on their religious leaders, and cultural influences.

## Barriers Faced by Jews

A review of existing literature on barriers faced by Jews to initiating psychotherapy resulted in the identification of the following possible barriers: reliance on Judaism, Rabbinical influence, cultural influence, and stigma.

### Religious devotion

A common belief in Judaism is that difficulties Jewish people experience are religious tests from God, and devout Jews should be able to pass without outside help (Sublette & Trappler, 2000). Schnall (2006) found that Orthodox Jews thought seeking psychological help indicated personal weakness and loss of faith in their religion. Rabinowitz (2000) noted that Orthodox Jews tended to believe that if they felt anxious, then they were not devout enough. Rabinowitz also wrote that a Jew seeking therapy might prefer to seek out a non-Jewish therapist familiar with and supportive of Judaism instead of a nonreligious, secular Jewish therapist who might impose a secular view.

Loewenthal (2006) mentioned Yichud as a possible barrier for psychotherapy. Yichud prohibits men and women from being alone together, meaning that the therapist would not be able to see an Orthodox Jewish client of the opposite gender without a chaperone. Yichud also states that a Jew must call the most competent doctor regardless of religion or culture. Additionally, the same law states that a Jew must not give their Jewish child to a non-Jewish teacher. Loewenthal (2006) found that most interpretations put a psychotherapist in the teacher category, which might create a barrier to psychotherapy.

### Trust in the Rabbi

Religious Jews often preferred to resolve issues by going to their Rabbi (Miller & Lovinger, 2000). Rabinowitz (2000) noted that some Jews prefer going to Rabbis because Rabbis often give direct answers to their question. However, a Rabbi’s referral to a psychotherapist may carry a lot of weight and could be the only way to persuade an Orthodox Jew to come to therapy. (Schnall, 2006). Without a Rabbi supporting a therapist, the Rabbi’s congregation might not go to therapy, thus leading Rabbinical influence to becoming a barrier to therapy.

### Jewish Culture

Jewish community is typically an important part of Jewish culture (Rabinowitz, 2000). The Orthodox Jewish community is particularly close-knit. Consequently, Orthodox clients may worry that others would find out about attending therapy, which would hurt their family’s status and marriage prospects (Schnall, 2006). In their qualitative study of providers who treated Orthodox Jews in London, Loewenthal and Rogers (2004) found that Jews reported feeling supported by other Jewish people and believed that non-Jews would not be able to understand their problems. Without therapists competent in the Jewish culture or religion, the Jewish person will be more inclined to solve their problems by following cultural and religious values than seeking out therapy.

Furthermore, in a 2021 survey by Pew Research Center, 27% of Jewish Americans in the United States identified as culturally or ethnically Jewish and not religiously Jewish (Alper & Cooperman, 2011). Due to the complex nature of Judaism and the need for research in general on Judaism, this study focused on participants who identified as “Jewish” and then ascertained what that meant to them through the demographic questionnaire.

### Stigma

Schnall, (2006) found that seeking psychological help was seen as weakness among many Jews, which potentially creates a barrier to seeking psychological help. What is wrong with the modern Jews that they cannot solve their problems without going to therapy when the older generations survived so much without therapy?

In her own literature review to identify barriers London Jews faced when seeking psychological help, Loewenthal (2006) found several studies indicating that many minority religious and ethnic groups viewed using mental health services as stigmatizing for the family and other members of the groups. She suggested stigma as one barrier to seeking mental health services.

## Gap in the Literature

As mentioned throughout this review, few studies have directly investigated the link between many sects of Judaism and seeking therapy. The closest article or study the researchers could find about the barriers to initiating therapy was Loewenthal and Rogers’ (2004) study, and Loewenthal’s (2006) proposed barriers to therapy. Although very similar to the focus of this study, Loewenthal and Rogers (2004) focused only on a small Orthodox Jewish community in London with a small sample size and did not focus solely on psychotherapy services. The data findings might be difficult to generalize to other communities. Loewenthal (2006) did provide a good foundation as to how to tailor the current study. This study looked at Jews from all sects of Judaism, with varying degrees of religiosity or cultural attunement, to fill the gap needed for research on barriers for Jews to seeking out therapy.

## Hypotheses

Researchers have not evaluated specific factors preventing Jews from initiating therapy. This study filled the gap in the literature by directly surveying Jews about possible barriers to engaging in therapy. This study investigated the influence of religiosity, Jewish culture, Rabbinical influence, stigma, and sect of Judaism as possible barriers to therapy by Jews. The researchers hypothesized that:

1. The more religious Jewish people are, the more negative attitudes they will have toward seeking psychotherapy.
2. The more culturally identified Jews are, the more negative attitudes they will have toward seeking psychotherapy.
3. The more Jews trust their Rabbi, the more negative attitudes they will have toward seeking psychotherapy.
4. The more Jews stigmatize psychotherapy, the more negative attitudes they will have toward seeking psychotherapy.
5. Sect of Judaism will influence each of the predictor variables (religiosity, cultural identity, trust in Rabbi, and stigmata) and the outcome variable attitudes toward seeking psychotherapy.

## Methods

The study used a cross sectional survey design to collect data on Jewish American attitudes toward initiating psychotherapy. This study focused on participants who identified as “Jewish,” with the term “Jew” as a common reference to one who identifies with Judaism religiously or culturally or both. The study was correlational and used primary data collected by the primary author after university IRB approval. The data were analyzed through descriptive statistics, multiple linear regression analysis, and ANOVAs with post hoc analyses using the LSD post hoc criterion for significance.

### Demographics of Sample

Inclusion criteria of the sample were Jewish-identified individuals over the age of 18 living in the U.S.A. A total of twenty percent of the sample was between the ages of 18 and 24, 40% was between 35 and 45, and 40% was above 45 years old. Then, sixty-one percent of the sample was female. Finally, forty percent identified as Reform, 31% Conservative, 14% Jew-Other, 11% Orthodox and 4% identified as cultural Jews only. Please refer to the Appendix for a more comprehensive summary of sample demographics.

### Procedure

The primary researcher recruited participants via online flyer, through networking with the San Diego Jewish community, and using contacts of leaders of other Hillels and communities outside of San Diego. Emails containing the flyer were sent to three Hillels, synagogues or Jewish Family Services in the continuous United States in order to obtain as much of a comprehensive sampling of religious and cultural and combination thereof of Jews for this study. Previous research has indicated a lack of generalizability in research on Jews, and this sampling attempted to combat that. The link was also posted in social media outlets. The flyer detailed eligibility requirements.

Once recruited, participants accessed the online survey, read the informed consent, and then checked a box to indicate their consent and eligibility requirements. Then the survey directed the participants to complete the measures explained below. After preliminary analysis where the primary researcher manually confirmed participants’ eligibilities, of the 138 submitted surveys, only 124 fulfilled the Jewish identity requirement, and only 94 of those surveys were fully completed and eligible for analysis. An a priori power analysis using G*Power software (3.1) indicated that a minimum sample size of 85 participants would be necessary for a multiple regression to generate a medium effect size of 0.06 (*r*^2^), a power of 0.8 (appropriate for social sciences), while using a critical alpha of 0.05. Thus, even though a small sample size, this study’s participant sample was large enough for analysis. We discuss limitations in a later section.

The study used the Centrality of Religiosity Scale which is a 10-item Likert scale that measures how religious a person considers themselves to be (Religiosity; Cronbach’s *α* = 0.93); American Jewish Identity Scale, which is a 15-item Likert scale that measures how culturally Jewish a person considers themselves to be (Culture Scale; Cronbach’s *α* = 0.88); Leadership Scale, which is a three-item Likert scale that measures how much a person trusts a leader (Trust; Cronbach’s *α* = 0.72), Perceived Public Stigma scale, which is an 11-item Likert scale that measures the stigma a person perceives (Stigma; Cronbach’s *α* = 0.89); and Attitudes Toward Seeking Professional Psychological Help, which is a 10-item Likert scale that measures the attitudes a person has toward seeking out psychotherapy (Attitudes Scale; Cronbach’s *α* = 0.84).

## Results

### Hypotheses One to Four

Stepwise multiple regression was used to test hypotheses one–four. No multiple regression assumptions were violated. The researcher performed a stepwise regression analysis using SPSS to determine the significant predictor variables on the dependent variable (Table 1).

**Table 1:** Summary of multiple linear regression analysis for variables predicting attitudes toward seeking psychotherapy

Variable	Standardized beta	Unstandardized beta	*t*	*R*^2^ change (*p* value)	*F* change (*df*)	Adjusted *R*^2^
Model 1				0.063	6.204	0.053
Stigma scale	−0.251	−2.113	−2.49	(0.015)	(1, 92)	
Excluded variables						
Religiosity			0.039	(0.969)		
Culture scale			1.224	(0.224)		
Trust scale			0.974	(0.333)		

There was no significant association between Religiosity and Attitudes (*p* = 0.969), between Culture and Attitudes (*p* = 0.224), or between Trust and Attitudes (*p* = 0.333; Table 1). However**,** there was a significant association between Stigma and Attitudes (*p* = 0.015) with a PCC of −0.214. This analysis indicate that stigma perceived by Jewish people was the only variable that predicted Jewish people’s attitude toward initiating psychotherapy (Table 1).

### Hypothesis Five

The first ANOVA showed sect of Judaism had a significant effect on Religiosity, *F*(3,89) = 7.361, *p* < 0.001. Post hoc analyses showed significant differences between: Orthodox Jews and Jew-Others (*p* = *0.0*47); Orthodox and Conservative Jews (*p* = 0.003); Conservative and Reform Jews (*p* < *0.0*01); and Reform Jews and Jew-Others (*p* = 0.023; Table 5).

The second ANOVA showed that sect of Judaism had a significant effect on Culture, *F*(3,89) = 3.540, *p* = 0.018. Post hoc analyses revealed differences between Conservative Jews and Reform Jews (*p* = 0.002; Table 2). An ANOVA showed that sect of Judaism had a significant effect on Trust, *F*(3, 89) = 3.324, *p* = 0.023. Post hoc analyses revealed differences between Jew-Others and Reform Jews (*p* = 0.032), and between Conservative Jews and Reform Jews (*p* = *0.0*04; Table 2).

**Table 2:** ANOVA effect of sect of Judaism on predictor variables and outcome variable

Variable	Orthodox		Conservative		Reform		Jew-other	
	*M*	*SD*	*M*	*SD*	*M*	*SD*	*M*	*SD*
Religiosity***	35.10	9.78	26.36	7.37	34.51	6.59	28.38	10.36
Culture scale**	43.50	7.68	38.26	8.52	45.51	9.52	42.08	11.47
Trust scale*	4.30	1.22	3.68	1.64	4.67	1.12	3.67	1.21
Stigma scale	2.46	0.79	2.34	0.75	2.36	0.58	2.46	0.74
Attitudes scale	23.80	4.29	24.55	4.42	23.72	5.70	21.61	8.11

*p < 0.05; **p < 0.01, ***p < 0.001

### Additional Analysis

ANOVAs showed that location, relationship status, age and gender had significant effects on the predictor and outcome variables (Table 3).

**Table 3:** ANOVA effect of location on predictor variables and outcome variable

Variable	East		West		Mid/South/North	
	*M*	*SD*	*M*	*SD*	*M*	*SD*
ANOVA effect of location on predictor variables and outcome variable						
Religiosity	31.95	9.73	30.11	8.55	26.67	6.95
AJIS	42.46	9.35	40.15	10.69	43.50	5.96
CLS	3.99	1.50	4.08	1.46	4.39	0.85
Stigma scale	2.40	0.61	2.38	0.71	2.35	0.59
Attitudes scale*	21.91	6.50	23.64	5.37	28.50	1.38

Variable	Unmarried/not cohab		Unmarried/cohab		Married/cohab	
	*M*	*SD*	*M*	*SD*	*M*	*SD*
*ANOVA effect of relationship status on predictor variables and outcome variable*						
Religiosity	31.53	8.89	25.91	6.83	30.56	9.03
AJIS	40.21	9.72	39.08	6.91	42.15	11.27
CLS	4.36	0.97	4.36	1.68	3.73	1.67
Stigma scale**	2.65	0.61	2.13	0.47	2.23	0.71
Attitudes scale	22.47	6.43	23.50	5.00	24.22	5.26

Variable	18–24 years old		25–35 years old		35–45 years old		45–55 years old		55–65 years old		65 years old or older	
	*M*	*SD*	*M*	*SD*	*M*	*SD*	*M*	*SD*	*M*	*SD*	*M*	*SD*
*ANOVA effect of age on predictor variables and outcome variable*												
Religiosity***	30.89	6.93	29.72	8.72	28.71	9.67	32.83	10.89	37.2	7.33	24.5	8.00
AJIS*	40.26	10.07	40.81	9.44	33.00	11.11	50.00	6.45	48.5	5.94	41.0	7.52
CLS	4.39	1.01	4.54	1.03	3.02	2.06	3.83	1.50	3.77	1.60	3.63	1.50
Stigma scale	2.36	0.76	2.52	0.63	2.02	0.76	2.62	0.55	2.26	0.44	2.41	0.74
Attitudes scale*	20.73	7.13	24.57	4.57	25.51	2.79	21.33	5.92	24.7	3.77	21.5	9.32

*p < 0.05; **p < 0.01, ***p < 0.001

### Second Regression Model

Due to the significance of ANOVAs, the researcher ran a second regression to determine other predictor variables for Attitudes. A stepwise multiple linear regression was run with the hypothesized predictor variables, sects of Judaism, location, relationship status, age, and gender as predictors (Table 4). Categorical variables were dummy coded to be used in a regression. Male gender (*Beta* = −0.564, *t*(84) = 6.257, *p* < 0.001), and Trust (*Beta* = 0.185, *t*(84) = 2.049, *p* = 0.044) significantly predicted attitudes toward psychotherapy (Table 4). Once gender is added to the regression model, stigma was no longer significant.

**Table 4:** Summary of second multiple linear regression predicting attitudes toward psychotherapy

Variable	Standardized beta	Unstandardized beta	*t*	*R*^2^ change (*p* value)	*F* change (*df*)	Sig. *F* change	Adjusted *R*^2^
Model 2				0.034	4.200 (1,84)	0.044	0.311
Male	−0.564	−6.330	6.257	(0.000)			
Trust scale	0.185	0.701	2.049	(0.044)			

### Correlations of Second Regression: Table 5

Attitudes negatively correlated with Stigma, (*p* = 0.008), Jew-others, (*p* = 0.022); and males, (*p* < 0.000). Culture positively correlated with Religiosity (*p* < 0.000), with Reform Jews (*p* = 0.006), and negatively correlated with Conservative Jews (*p* = 0.001). Trust positively correlated with Religiosity (*p* = 0.020) and Reform Jews (*p* = 0.005). Trust negatively correlated with Conservative Jews (*p* = 0.013). Stigma positively correlated with males (*p* = 0.019). Stigma negatively correlated with Attitudes (*p* = 0.008). Religiosity positively correlated with Culture (*p* < 0.000), Orthodox Jews (*p* = 0.031), and Reform Jews (*p* = 0.001). Religiosity negatively correlated with Conservative Jews (*p* < 0.000).

**Table 5:** Pearson correlations of second multiple regression

	Attitudes Scale	Culture Scale	Trust Scale	Stigma Scale	Religiosity	Age
Attitudes scale	1.000	0.029	0.119	−0.256*	−0.006	−0.016
Culture scale	0.029	1.000	0.161	0.049	0.600	0.102
Trust scale	0.119	0.161	1.000	0.053	0.221	−0.217*
Stigma scale	−0.256*	0.049	0.053	1.000	0.077	−0.036
Religiosity	−0.006	0.600*	0.221*	0.077*	1.000	0.013
Age	−0.016	0.102	−0.217*	−0.036*	0.013	1.000
Orthodox	0.009	0.067	0.057	0.038	0.201	−0.162
Conservative	0.142	−0.320*	−0.239*	−0.054	−0.392	0.095
Reform	−0.003	0.271*	0.278*	−0.050	0.343	−0.102
Jew-other	−0.216	0.032	−0.090	0.114	−0.090	0.158
Male	−0.542*	0.062	0.117	0.222*	0.144	0.013

	Age	Orthodox	Conservative	Reform	Jew-Other	Male
Attitudes scale	−0.016	0.009	0.142	−0.003	−0.216*	−0.542*
Culture scale	0.102	0.067	−0.320*	0.271*	0.032	0.062
Trust scale	0.217*	0.057	−0.239*	0.278*	−0.090	0.117
Stigma scale	−0.036	0.038	−0.054	−0.050	0.114	0.222*
Religiosity	0.013	0.201*	−0.392*	0.343*	−0.090	0.144
Age	1.000	−0.162	0.095	−0.102	0.158	0.013
Orthodox	−0.162	1.000	−0.317*	−0.248*	−0.137	0.145
Conservative	0.095	−0.317*	1.000	−0.607*	−0.335*	−0.155
Reform	−0.102	−0.248*	−0.607*	1.000	−0.262*	−0.013
Jew-other	0.158	−0.137	−0.335*	−0.262*	1.000	0.111
Male	0.013	0.145	−0.155	−0.013	0.111	1.000

*Significance *p* < 0.05 (1–tailed); *n* = 87

## Discussion

A Jew’s religiosity, culture, or trust in a Rabbi were not found to be significantly related to Attitudes on initiating psychotherapy (Hypotheses One–Three). However, Stigma was found to be significantly related to Attitudes. The more stigma Jews perceived about therapy, the less likely they would want to initiate therapy (Hypothesis Four). Sect of Judaism (Orthodox, Reform, Conservative, and Other) did not have a significant effect on Attitudes (Hypothesis Five). This is inconsistent with previous research (Feinberg & Feinberg, 1985; Baruch et al., 2014) that suggests that Orthodox Jews have more negative views of seeking psychotherapy than other sects of Judaism. This may be due to sample size, or outdated research as these results indicated that attitudes regarding seeking psychotherapy may be changing in the Orthodox Jewish community. These data suggest that attitudes toward psychotherapy among the sects of Judaism may be changing, so more research is necessary (Schnall et al., 2014). Since this data set cannot truly be generalizable to all Jewish people in the U.S. (see the Limitations section), it should be used as information for future studies seeking to find more results about Jewish help-seeking behaviors.

However, using ANOVAs, the sect of Judaism with this sample was found to be related to Religiosity, Culture, and Trust. In this sample, Orthodox Jews scored higher on religiosity, meaning that they consider themselves more religious, than Jew-Others and Conservative Jews. Conservative Jews scored lower, meaning that they consider themselves less religious, than Reform Jews. Reform Jews scored higher, meaning that they consider themselves more religious, than Jew-Others. Conservative Jews and Jew-Others had similar averages. Conservative Judaism attempts to find a middle ground for most Jews (Miller & Lovinger, 2000); Jew-Others might also be trying to find a balance of beliefs and values, so might explain the similar scores. Reform Jews scored similar to Orthodox Jews. Though Reform was founded as a reaction to Orthodox, they have firm beliefs in their religion (Miller & Lovinger, 2000).

Reform Jews scored significantly higher on Culture and Trust than Conservative Jews in this sample. Therefore, Reform Jews consider themselves more culturally Jewish than Conservative Jews. This is consistent with reports that Reform Jews tend to have a strong focus on the traditions surrounding Judaism (Sheshkin & Hartman, 2019). Moreover, Jewish culture contains many rituals. If the sects of Judaism were considered on a continuum of adherence to ritual, Orthodox Jews would be the strictest, then Reform Jews, then Conservative Jews, then Jew-Others (Sheshkin & Hartman, 2019). This same process may explain the results of this study that indicate Reform Jews trust their Rabbis more than Conservative Jews or Jew-Others. This may be because the Rabbi is an integral part to the culture and adherence to rituals.

### Location, Age, Relationship Status, and Gender

#### Location

Participants who grew up on the East and West Coasts scored lower on Attitudes than those who grew up in the Midwest. This means that participants from the East and West Coasts reported being less likely to initiate therapy than those from the Midwest. Orthodox Jews typically reside in those areas and may have affected the data with more negative views about going to therapy. Sample size could account for this result as well, as only six participants were from the Midwest, resulting in a possible Type I error. Most Jews grew up on the coasts because many immigrated thereafter or during WWII.

#### Age

Participants’ age had a significant effect on Religiosity, Culture and Trust. Participants between 55 and 65 y.o. scored higher on Religiosity, meaning that they consider themselves more religious, than 25–35 y.o. and 35–45 y.o. Many older Jews are usually Orthodox or Reform, which are more religious sects (Sheshkin & Hartman, 2019). Older Jews may be firmer in their religious beliefs than younger Jews.

The 55–65 y.o. reported themselves as more culturally Jewish than 18–24 y.o. and 65 y.o. and older. Eighteen to 24 y.o. considered themselves more culturally Jewish than 35–45 y.o. This may be due to the founding of Birthright Israel in 1999 (Birthright; Taglit, 2020). Birthright is a free trip offered to Jews 18–26 y.o. to strengthen Jewish identity through cultural immersion in Israel. Thus, younger Jews may be contemplating Birthright or have already made the trip. Their Jewish cultural identity may be more poignant at their time in life than 35–45 y.o.

Finally, 35–45 y.o. report less trust in their Rabbi than do 18–24 y.o. or 25–35 y.o. This may be due to 35–45 y.o. no longer wanting guidance from their Rabbis, as they may have clearer life goals or a general direction for their life. College-bound or quarter-life crisis participants may need spiritual guidance to help them through tough times.

#### Relationship Status

A participant’s relationship status had a significant effect on Stigma. Single participants scored higher on Stigma than both the cohabitating and the married participants in this study. Cohabitating people may be more open to therapy: They challenge the belief about pre-marital cohabitating, so they may challenge the stigma of therapy (Walsh, 2010).

#### Gender

Gender had a significant effect on Stigma and Attitudes. Female participants scored lower on Stigma than male participants, indicating that females had less stigma to psychotherapy, consistent with findings on the general population (Lannin et al., 2016; Liddon et al., 2018). Males may have the sense that they need to be the protectors of the family and faith, and the heads of the family. Therefore, they would not want to seek mental health services, as that would imply a failure as the head of household (Brenner et al., 2018).

### Second Regression Model

When Gender was included in the second stepwise multiple regression, stigma was no longer significant, and trust was significant. People who have high trust in their Rabbi are also more likely to have positive attitudes about seeking psychotherapy. This would suggest that there is a strong correlation between stigma and gender, with Jewish males having more stigma. When that is taken into account, the trust Jews have in their leadership becomes an important factor for seeking out therapy, particularly among males.

### Correlations

When age was used as a continuous scale variable, it was found, in this study, that the older participants are, the less likely they are to trust their Rabbi, the less stigma they have against therapy, and the more likely they are to be married. Older persons generally are more open to psychotherapy due in part to Medicare coverage (Knight, 2009). Older Jews might not have as much trust in the Rabbi because they may realize that the Rabbi might not know as much as previously thought. The older the Jew, the more likely they are to be married and living together because Jews are taught the value of family and marriage and are taught to find a spouse. It is also frowned upon in the religion (especially those following Orthodoxy) for a couple to live together while not married (Birenbaum-Carmeli et al., 2021). The positive correlation between Culture and Religiosity may be because religious Jews consider themselves culturally Jewish as well.

### Clinical Implications

Since Jews’ trust in their Rabbi is positively correlated with initiating psychotherapy, Rabbis may be able to influence them to seek out therapy, especially when it comes to males. This is significant given that men often do not seek out therapy (Brenner et al., 2018). It may be important for clinicians to establish relationships with Rabbis to encourage Rabbis to refer to therapy members in their congregation. Since age and sect of Judaism both have significant differences on Trust, clinicians should be aware that Jews between 35 and 45 y.o. are less influenced by their Rabbi. If Rabbis are to influence this group, they will likely need to develop more trust with them. Clinicians should also note that Conservative Jews and Jew-Others might need more rapport with their Rabbis if the Rabbi is to influence them to go to therapy.

## Limitations

Although small, the Jewish community is very diverse: There are different sects of Judaism, different denominations within the sects, Jewish people from different countries of origin, different levels of acculturation by living in the United States, etc. To group all of the Jews together means the results may not be valid for specific subgroups of Jewish people in the United States.

The researcher bracketed age groups incorrectly, (e.g., 25–35 y.o. and 35–45 y.o.), thus contributing to error. Furthermore, the majority of participants were younger than 45 y.o. which could have influenced the predictor variables. It could be that older Jews are less influenced by Rabbis or religion, but there may not be enough participants in that demographic of this sample to determine that for certain. There could be a myriad of reasons for the difference in numbers of participants in age groups: One reason could be that older Jews do not want to be disrupted by an online survey, and they may not know how to access an online survey.

The dropout rate is a limitation because 24.2% of eligible participants did not complete all measures. The researcher is unable to ascertain a reason through analysis, or if there were any patterns. It should be further noted that these data are correlational and therefore direction of influence is theoretical and not causal. However, as mentioned earlier, during the initial a priori analysis to determine appropriate sample size, the researcher found the adequate minimum sample size to be 85 participants. Even though this study fulfilled the minimum sample size, larger sample sizes are always preferred in order to provide more robust and accurate results.

Another limitation is the use of the survey itself without a pilot study. Without a pilot study to test the reaction to the created survey, there may be unanticipated reactions that might lead to attrition. As such, the researcher was not be able to determine if there is a length of surveys that would be more appropriate for the study. For example, during analysis, attrition might occur after a certain number of items in the study; a pilot study would aid in discovering a better way of structuring the measures.

### Future Directions

This initial study’s benefit of focusing on the Jewish population as a whole in regard to barriers to psychotherapy will allow for more specific research to occur. Weinrach (2002) mentioned the lack of Jewish research within the psychotherapy realm; the results of this study will help fill that gap. This study is a stepping stone for future studies.

Future researchers should focus on how to get males in to therapy, particularly if they do not have much trust in their religious leader. Future researchers may find it helpful to include a broader sample of Jews in order to make the research more generalizable, as well as a more detailed analysis of the sect and denomination of the participants rather than just dividing them into four broad categories. More research is needed about how location could influence a Jew’s perspective on initiating psychotherapy. Additionally, qualitative analysis where Jewish people are interviewed regarding their perception of barriers to psychotherapy could be helpful.

## Appendix

### Participant Demographics Characteristics

**Table:**

Variables	*N*	%
Age		
18–24 years old	21	16.9%
25–45 years old	69	55.6%
45–65 years and older	34	27.5%
Income		
Under $25,000	8	6.5%
$25,000–34,999	7	5.6%
$35,000–49,999	13	10.5%
$50,000–74,999	14	11.3%
$75,000–99,999	22	17.7%
$100,000–149,999	16	12.9%
$150,000–199,999	16	12.9%
$200,000 or more	27	21.8%
Gender		
Female	79	35.8%
Male	44	64.2%
Education—highest level		
High school graduate	12	9.7%
College graduate	41	33.1%
Post graduate	71	57.3%
Ethnic group		
White	112	90.3%
Hispanic/Latinx	2	1.6%
Other	9	7.3%
Religious identification		
Jewish	116	93.5%
Non-Jewish	5	4%
Other	3	2.4%
Denomination of Judaism		
Orthodox	11	8.9%
Reform	56	45.2%
Conservative	31	25.0%
Jew-Other	19	15.3%
Non-Jewish	7	5.6%
Father’s religious identity		
N/a	3	2.4%
Nonreligious secular	3	2.4%
Agnostic	6	4.8%
Atheist	1	0.8%
Judaism	99	79.8%
Catholicism	10	8.1%
Other	2	1.6%
Mother’s religious identity		
N/a	0	0%
Nonreligious secular	6	4.8%
Agnostic	2	1.6%
Atheist	5	4.0%
Judaism	100	80.6%
Buddhism	2	1.6%
Catholicism	7	5.6%
Other	2	1.6%
Parents in an inter-faith marriage (Jewish–Non-Jewish)		
Yes	26	21%
No	98	79%
Relationship status		
Unmarried, not cohabiting	40	32.3%
Unmarried, cohabitating	19	15.3%
Married, not cohabitating	1	0.8%
Married, cohabitating	61	49.2%
In non-monogamous relationship	3	2.4%
Location		
East coast, USA	26	21%
West coast, USA	84	67.7%
Midwest/south/north, USA	14	11.3%
