# Correction to: Barriers to Initiating Psychotherapy Faced by Jewish-Identified People in the United States

**DOI:** 10.1007/s10943-024-02115-3

**Published:** 2024-09-04

**Authors:** Anna Jean Berman, Scott Woolley

**Affiliations:** Alliant International University, 10455 Pomerado Road, San Diego, CA 92131 USA

**Correction to: Journal of Religion and Health** 10.1007/s10943-024-02097-2

In this article, the University Address in the affiliation section for the Authors was incorrect. The correct address information is ‘10455 Pomerado Road, San Diego, CA 92131, USA’.

The original article has been corrected.

